# Optimized Soft Lithography Method for Polymer Cholesteric Liquid Crystal Flakes Fabrication

**DOI:** 10.3390/mi10070441

**Published:** 2019-07-01

**Authors:** Guanqing Zhou, Sunqian Liu, Wei Liu, Dong Yuan, Guofu Zhou

**Affiliations:** 1 Guangdong Provincial Key Laboratory of Optical Information Materials and Technology & Institute of Electronic Paper Displays, South China Academy of Advanced Optoelectronics, South China Normal University, Guangzhou 510006, China; 2 SCNU-TUE Joint Lab of Device Integrated Responsive Materials (DIRM), National Center for International Research on Green Optoelectronics, South China Normal University, No 378, West Waihuan Road, Guangzhou Higher Education Mega Center, Guangzhou 510006, China; 3 Department of Physics and Astronomy and Collaborative Innovation Center of IFSA (CICFSA), Shanghai Jiao Tong University, Shanghai 200240, China

**Keywords:** soft lithography, polymer cholesteric liquid crystal, flakes, polyvinyl alcohol layer, release time

## Abstract

Polymer cholesteric liquid crystal (PCLC) flakes are gaining increasing interest for a wide variety of applications because of their unique optical properties and capabilities. Soft lithography is the most effective way to fabricate regularly shaped PCLC flakes. However, it is not easy to peel the flakes from the mold without breaking them. In order to peel the PCLC flakes from the patterned polydimethylsiloxane (PDMS) mold in a convenient way, in this paper, a method of coating a layer of polyvinyl alcohol (PVA) on a PDMS mold was proposed. The influence of the thickness of the PVA layer on the shape of the PCLC flakes and the release time from the PDMS mold were investigated. The results show that the presence of the PVA layer can speed up the release of the PCLC flakes and help maintain the shape effectively. Notably, the utilization of a PVA layer makes the PDMS mold recyclable. The influence of PCLC flake shape was also studied. This work will promote the development of switchable PCLC flake-based technologies.

## 1. Introduction

Polymer cholesteric liquid crystals (PCLCs) exhibit unique wavelength and polarization selective reflection owing to their molecular structure which is ordered in layers and rotates around an axis normal to the layers [1]. Because the glass-transition temperatures are well above room temperature, PCLCs can be manufactured as stable films, flakes or particles [2,3,4].

Switchable PCLC flake and particle-based technologies are gaining increasing interest for a wide variety of applications because of their unique optical properties and capabilities. An obvious application for switchable flakes is reflective displays which require low power consumption and have a paper-like visual appearance due to the use of ambient light. The reflective display (e-paper) market is predicted to grow dramatically in the coming years as it moves into applications which are currently dominated by traditional paper. PCLC flake technology for reflective display uses flakes for reflection, which is similar to electrophoretic particles (EPD), but based on the unique reflective properties of PCLC flakes [1,2]. PCLC flakes can also be used in infrared reflective device. The alignment direction of PCLC flakes in the device is driven by the rotation of a host liquid crystal in an electric field, which realize the switch from the infrared reflection state to the transmission state [5].

To develop commercially switchable flake-based technologies, one of the key factors is the fabrication of PCLC flakes. Several methods have been proposed over the past decades [5,6,7]. PCLC flakes have been produced by freeze-fracturing a PCLC film to break it down into irregularly shaped flakes with a length in the range of tens to hundreds of microns [8]. Irregular PCLC flakes can also be fabricated by ultrasonic crushing [5]. However, these two methods are not that effective and consume additional energy. In order to obtain well-defined and shaped PCLC flakes, a soft lithography method using a patterned polydimethylsiloxane (PDMS) mold is commonly used. Trajkovska-Petkoska fabricated shaped PCLC flakes using soft lithography and found that the shaped flakes were smooth and uniform in size, shape and thickness [9]. Trajkovska-Petkoska also found that PCLC flakes exhibit unique optical properties, such as selective reflection and circular polarization of reflected light [10].

**Figure 1:**
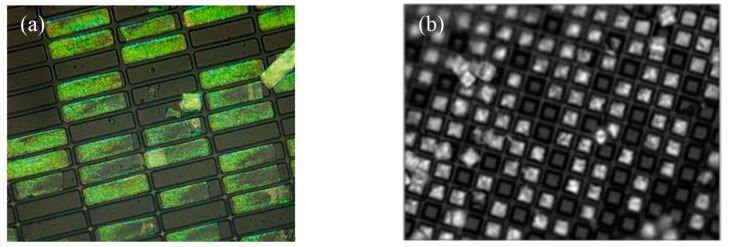
Flakes stick to the polydimethylsiloxane (PDMS) mold and cannot be peeled off: (**a**) Flakes fabricated by us without a polyvinyl alcohol (PVA) layer; (**b**) flakes in Trajkovska-Petkoska’s paper [9], Copyright 2005, John Wiley and Sons.

However, it is not easy to peel the PCLC flakes from the PMDS mold without breaking it during soft lithography fabrication. From Figure 1 we can see that the flakes might stick to the PDMS mold which will lead to problems upon release of the flakes. Moreover, in Trajkovska-Petkoska’s work, they quenched the PDMS mold and could not peel the all flakes away, as some of the flakes adhered to the PDMS mold. Also quenching the PDMS mold may damage it. 

To overcome the shortcoming of soft lithography, in this paper, a method of coating a polyvinyl alcohol (PVA) layer on the PDMS mold was proposed, which helps to release the PCLC flake. The influence of the thickness of the PVA layer on the shape of the PCLC flakes and the release time from the PDMS mold were investigated. The result shows that the PVA layer can speed up the release of PCLC flakes and effectively help to maintain the shape. 

## 2. Materials and Methods

### 2.1. Materials and Preparation

**Scheme 1:**
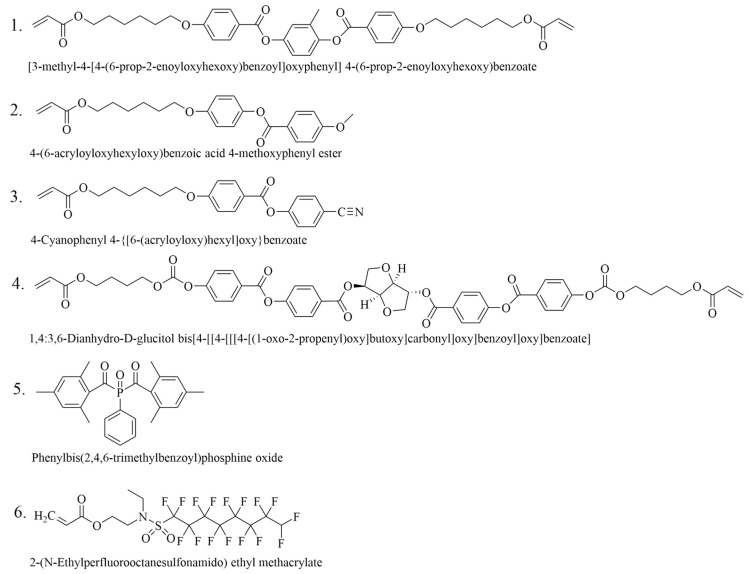
Chemical structure of the monomers used for the preparation of the polymer cholesteric liquid crystals flakes.

An overview of the materials used to fabricate the PCLC flakes is shown in Scheme 1. Monomers 1–4 were all obtained from Jiangsu Hecheng new materials Co. Ltd., Nanjing, China. Monomer 1 is a di-acrylate cross-linker. Monomers 2 and 3 are mono-acrylates. The phase transition temperature and mechanical strength of the mixed material change when the ratio between 1, 2 and 3 is varied. Monomer 4 is a chiral dopant which allows variation of the pitch lengths of PCLC by controlling its concentration. Photo-initiator 5 was obtained from BASF SE, Ludwigshafen, Germany, and was added to allow photo-initiation of the polymerization by exposure to UV light. Monomer 6 is a surfactant which induces planar alignment at the interface of the liquid crystal (LC) material and air. It was obtained from Shenzhen Regent Biochemistry Technology Co. Ltd., Shenzhen, China. Typically, the liquid crystal mixtures were fabricated from monomer 1, monomer 2, monomer 3 (the proportion of these three monomers was 2:3:4), monomer 4 whose concentration will be determined by the target reflection band (about 3–4 wt.% in this paper), 2 wt.% of monomer 5, 0.5 wt.% of monomer 6, and 200 ppm of polymerization inhibitor.

The monomers were mixed by dissolving them in dichloromethane, which was subsequently evaporated. A Differential Scanning Calorimeter (DSC) result shows that the mixture was in the cholesteric phase in the temperature range between 40 °C and 65 °C, as shown in Figure S1 in the Supplementary Material. At higher temperatures it became isotropic.

### 2.2. PDMS Mold Manufacture

**Figure 2:**
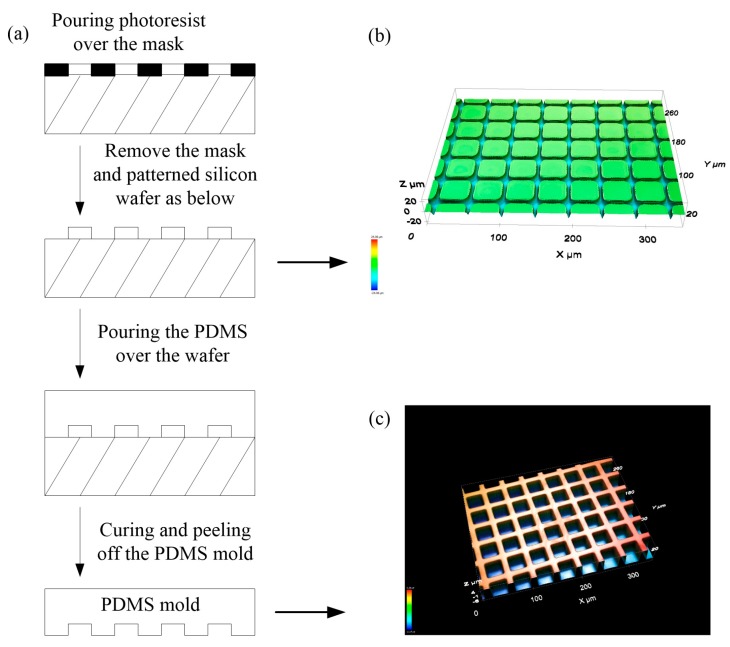
PDMS mold for soft lithograph: (**a**) A schematic illustration of the PDMS mold manufacturing process; (**b**) A patterned silicon wafer with a 10 μm high square pattern; (**c**) A PDMS mold made using the patterned silicon wafer shown in b.

Figure 2 shows the schematic diagram of the PDMS mold manufacturing process. In order to fabricate the well-defined PDMS mold, photo lithography was used to manufacture a patterned photoresist wafer. The detailed photo lithography process is as follows: A layer of photoresist (HN-008, Suntific Materials Co., Ldt., Weifang, China) was spin-coated over the glass substrate with 450 rpm rotation speed for 30 s. The photoresist was pre-baked at 110 °C for 5 min to make the photoresist adhere to the surface of glass substrate, and the sample was cooled down to the room temperature afterward. Exposure with mask on the lithography machine (URE-2000/35, Institute of Optics and Electronics, Chinese Academy Science, Chengdu, China) for 10 s, the intensity of UV light was 27 mw/cm^2^ and the wavelength was 365 nm. During exposure, the exposed part of photoresist was cross-linked. The photoresist was baked again at 110 °C for 5 min to ensure that the cross-linked part can stayed on the glass substrate after development. The sample was immersed in 0.4% KOH for 3 min to develop the patterns; the unexposed photoresist dissolved in the develop solution patterns were formed. The height/depth of the patterns on the PDMS mold was controlled by the thickness of the photoresist. A PDMS mixture was poured over the photoresist wafer section, allowed to cure for 1 h at 60 °C, and then peeled off. The patterns on PDMS molds used in this paper had 4 different sizes: 30 μm × 30 μm, 20 μm × 60 μm, 30 μm × 60 μm, and 60 μm × 60 μm.

### 2.3. The Manufacture of PCLC Flakes

**Figure 3:**
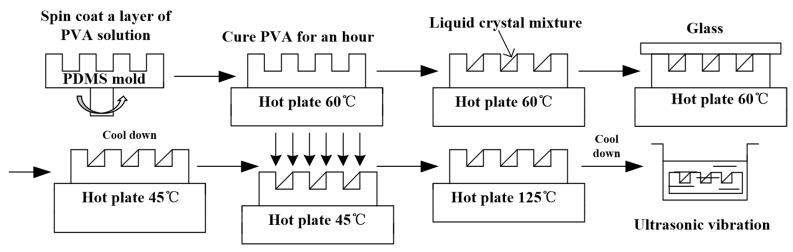
Fabrication process of polymer cholesteric liquid crystals (PCLC) flakes using soft lithography.

The method for manufacturing regularly shaped PCLC flakes is shown in Figure 3. The process flow is as follows: (a) The 5 wt.% PVA (MW:9000-10000, grade of hydrolysis: 80%, Sigma-Aldrich, St. Louis, MO, USA) was dissolved into water and stirred for 5 h at 50 °C, then the PVA solution was spin-coated onto the PDMS mold; (b) the PDMS mold was placed on a 60 °C hot plate for 1 h to dry the PVA; (c) the PDMS mold was filled with LC mixture and blade coating was applied to the LC mixture; (d) a glass plate with a rubbed parallel alignment layer was put over the PDMS mold containing the LC mixture and the glass was pushed in order to squeeze out the redundant LC mixture and align the LC molecules with the rubbed parallel alignment layer on the glass; (e) the sample was slowly cooled down on the hot plate to 45 °C, then the upper glass plate was removed and “knife-coating” was applied to the sample with the same glass plate; the sharing force of knife-coating was used to enhance the alignment of LC molecules; (f) the LC mixture was polymerized using a UV light source under nitrogen atmosphere at 45 °C for 5 min; the intensity of the UV light source (OmniCure S1500, EXFO Photonic Solutions Inc., Mississauga, ON, Canada) we used was 200 mw/cm^2^. The wavelength of the UV light was 320–500 nm; (g) post cure was achieved by heating at 125 °C for 15 min and then slowly cooled down to room temperature on the hot plate; (h) the sample was immersed in water and the ultrasonic vibration was used to release the PCLC flakes from the molds.

### 2.4. Characterization and Measurements

The phase transition temperatures of the mixtures were studied using Differential Scanning Calorimeter (DSC, METTLER DSC 1, Mettler Toledo, Inc., Zurich, Switzerland). The heating and cooling rates were both 2 °C/min. Optical images between crossed polarizers were taken using a polarized optical microscope (POM, Leica DM 2700 P, Leica Microsystems Ltd., Wetzlar, Germany). Transmittance spectra of the reflectors were measured using an Ocean Optics spectrometer 2000 PRO. The surface topography of PCLC flakes was measured using a 3D surface profilometer (Leica DCM8, Leica Microsystems Ltd., Wetzlar, Germany). In order to release PCLC flakes, we immersed the PDMS mold filled with flakes in water and shook it using the ultrasonic cleaner (SG3200HBT, Shanghai Guante ultrasonic instrument co., Ltd., Shanghai, China). The status of flake release was checked every minute. When there were no flakes left on the mold, the total ultrasonic time was recorded as the release time of the PCLC flakes. 

## 3. Results and Discussion

### 3.1. Characterization of Shaped Flakes

**Figure 4:**
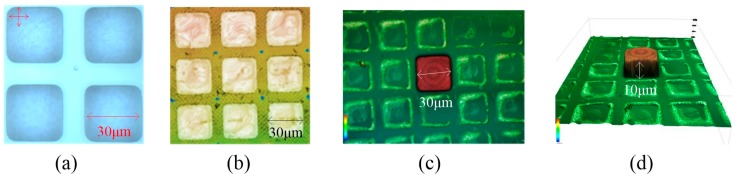
Characterization of flakes: (**a**) POM image of PCLC flakes in a PDMS mold; (**b**) The topography of PCLC flakes in PDMS mold; (**c**) the width of a single PCLC flake; (**d**) the height of a single PCLC flake.

The shape, alignment, and morphology of the PCLC flakes with 30 μm × 30 μm size in the PDMS mold with 0.5 μm PVA layer were studied, as shown in Figure 4. From the POM image of the flakes (Figure 4a), we can see that the PCLC flakes can copy the shape of the PDMS molds exactly. We can also see that the PCLC alignment inside the flake is not perfect; there are some dark dots that represent domains with different alignment directions, especially near the edges. This is because there is no alignment layer in the PDMS mold, but the LCs tend to align parallel at the interface with PDMS and PVA. This is conflicting with the rotational tendency of the cholesteric liquid crystals at the edges, so the alignment is not very uniform. When the size of flakes becomes larger, the influence of the edges becomes smaller and the alignment is better. In order to learn more about the morphology of the PCLC flakes, we used a 3D profilometer to observe the topography of the PCLC flakes. From Figure 4b, we can see that the major part of the PCLC flakes surface is flat, but there are a few irregular bumps. Figure 4c,d shows a PCLC flake adhering on the glass plate when we polymerized the PCLC flake without taking off the glass plate. It can be seen that there are some concentric circles on the top of the flake; this means the bottom of the PDMS mold is not entirely flat. However, as the height of the concentric circles is relatively low, it will not influence the performance of the flakes.

**Figure 5:**
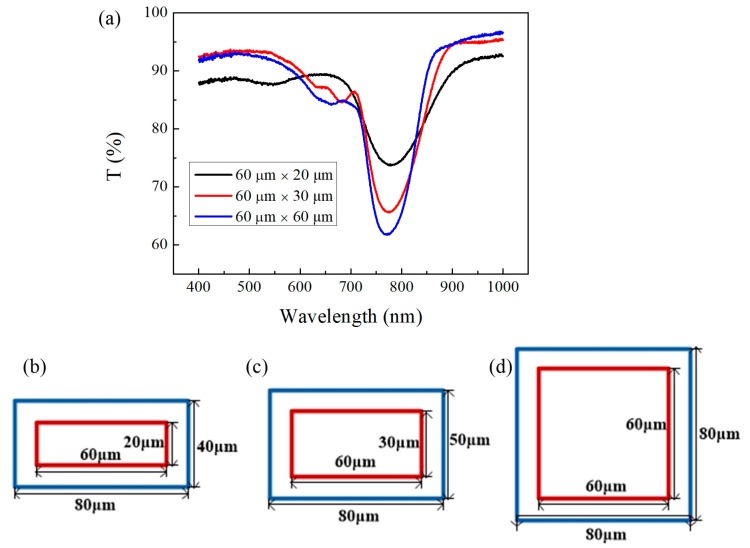
The transmittance and diagram of PCLC flakes in PDMS molds: (**a**) Transmittance of PCLC flakes in PDMS molds; (**b**) the diagram of the 60 μm × 20 μm size; (**c**) The diagram of the 60 μm × 30 μm size; (**d**) the diagram of the 60 μm × 60 μm size (The blue line means the PDMS mold and the red line means the PCLC flakes).

The reflectance of PCLC flakes with different aspect ratios (length/width) inside a PDMS mold is shown in Figure 5a. The aspect ratios of PCLC flakes are 1:1, 2:1, and 3:1 and the sizes of the flakes are 60 μm × 20 μm, 60 μm × 30 μm, and 60 μm × 60 μm, respectively. From Figure 5a, we can find that the reflectance of PCLC flakes at 760 nm wavelength is 15%, 22%, and 28%, respectively. The reflectance cannot reach 50% because the PCLC flakes only occupy a part of the whole area and they are too small to measure solely. More specifically, the size of light beam of the measurement instrument is much larger compared to the PCLC flakes. From Figure 5b–d, we can find that each size of PCLC flake occupies a different proportion of the whole area. The flake with a 3:1 aspect ratio occupies 37.5%, the flake with a 2:1 aspect ratio occupies 45% and the flake with a 1:1 aspect ratio occupies 56.25%. Since the reflection peak is only caused by the reflection of PCLC flakes and the PDMS mold has no contribution to it, the reflectance of the flakes can be calculated by the reflectance of PCLC flakes in a PDMS mold divided by the occupied area. Thus, the real transmittances of the PCLC flakes are 40%, 48.9% and 49.8%, respectively. So, the reflectance of larger PCLC flakes can reach the maximum value, which means the alignment inside the flakes is quite good. However, when the flake size is small, the alignment is influenced by the PDMS mold walls and the reflectance is lower. What is more, the irregular transmittance in the 600–700 nm wavelength range of Figure 5a is caused by the scattering of the PDMS mold.

### 3.2. Influence of PVA Layer Thickness

PVA is a water-soluble synthetic polymer. It is widely used in paper-making, textiles, and a variety of coatings [11]. It can also provide a parallel orientation of liquid crystals. Herein, in order to release the PCLC flakes from the PDMS mold more conveniently and keep the parallel orientation of the liquid crystals, we spin-coated a layer of PVA over the PDMS mold. Because different speeds of spin-coating will influence the thickness of the PVA layer and the depth of the PDMS mold, we used four spin-coating speeds to get different depths of the PDMS mold, including 500 rpm, 1000 rpm, 1500 rpm, and 2000 rpm. The spin-coat time was 30 s. We used the 60 μm × 60 μm PDMS mold to study the influence of the PVA layer thickness.

**Figure 6:**
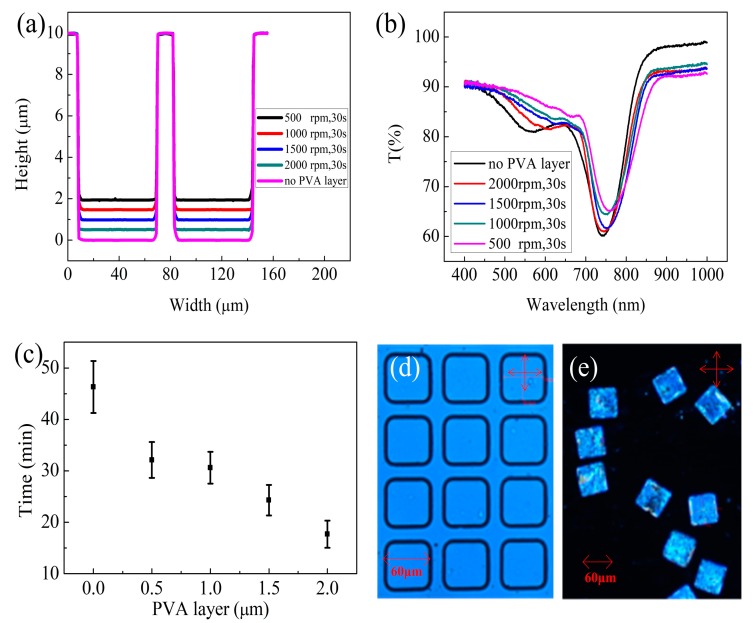
Influence of the PVA layer thickness on the reflectance and release time of the PCLC flakes from the 60 μm × 60 μm size PDMS mold: (**a**) Surface profiles of the PDMS mold after spin-coating PVA at different speeds; (**b**) transmittance of PCLC flakes with different thicknesses; (**c**) The release times of different thicknesses of PVA layers; (**d**) the POM image of the PDMS mold after flakes were released; (**e**) the POM image of the released PCLC flakes.

The depth of the PDMS mold after coating the PVA layer at different speeds can be seen from Figure 6a. We found that the PVA is mainly located at the bottom of the molds rather than at the walls. With the speed of spin-coating increasing, the PDMS mold becomes deeper because the PVA layer becomes thinner. The thickness of the PVA layer *h_f_* can be calculated by Equation (1):

(1) $$h_{f}={\left(\frac{3}{2}\right)}^{\frac{1}{3}}a_{0}{\left(1-a_{0}\right)}^{-\frac{1}{3}}\omega^{-\frac{2}{3}}\upsilon_{0}^{\frac{1}{3}}e^{\frac{1}{3}}$$

where *a*_0_ is the solute concentration of the PVA solution, *ω* is the angular speed, *υ*_0_ is the initial viscosity of the fluid, and *e* is the volatilization rate of the fluid [12,13,14,15]. Therefore, with the speed of spin-coating increasing, the *h_f_* of the PVA layer becomes thinner. Accordingly, the depth of the PDMS mold becomes deeper and the PCLC flakes become thicker. 

Furthermore, in order to study the influence of the PVA layer on the reflectance of the PCLC flakes, we tested the transmittance of the PCLC flakes in the PDMS mold with five different PVA thicknesses, as shown in Figure 6b. We can see that the reflectance of the PCLC flakes becomes lower when the spin-coating speed becomes lower, which means the PVA layer becomes thicker and the thickness of flakes becomes thinner. The reflectance *R* of a PCLC layer can be calculated by Equation (2) [16]:

(2) $$R=1-\exp\left(-\frac{2\pi \sigma \bar{n}d}{\lambda_{0}}\right)$$

(3) where, σ=[4(λ0n¯⋅P)2+(ne2−no2ne2+no2)2−1−(λ0n¯⋅P)2]12

(4) $$\bar{n}=\sqrt{\frac{n_{e}^{2}+n_{o}^{2}}{2}}$$

From the Equation (2) we can see that when the thickness *d* decreases, the reflectance also will decreases. With the thickest PVA layer, the reflectance of PCLC flakes in the PDMS mold is 24%; since the PCLC flakes only occupy 56.25% of the area in the PDMS molds, the reflectance of PCLC flakes is about 43.7%. When the PVA layer is thinner than 1 μm, the influence of the PVA layer on the reflectance of the PCLC flakes is very small, less than 2%. Therefore, the PVA layer influences the reflectance of the PCLC flakes because the flakes become thinner, but the influence is very small when the PVA layer is thinner than 1 μm.

The release time of the PCLC flakes in the PDMS mold with different PVA thicknesses is shown in Figure 6c. From this figure we can see that the release time of the flakes from a mold without a PVA layer is almost 50 min. Sometimes, there are still some flakes remaining inside the mold without a PVA after 1 h of ultrasonic vibration and the mold may be damaged. The releasing process is very time consuming and the PDMS mold could hardly be used again when a PVA layer was not used. After coating a PVA layer inside the molds, the release time drops significantly to less than 30 min and the release time becomes shorter when the PVA layer becomes thicker. When the PVA layer is 2 μm, the release time is only about 17 min. The PDMS mold (with 1 μm thick PVA layer) after release of the PCLC flakes is shown in Figure 6d. It shows that the PDMS molds can maintain their original shape and are unbroken. Since PVA can be dissolved in water, the mold can be reused after cleaning. The released PCLC flakes are shown in Figure 6e. The flakes are neat and uniform which is an improvement over the previous result. So coating a layer of PVA in the PDMS molds for PCLC flake fabrication using the soft lithography method can make the mold recyclable and save time.

### 3.3. Influence of Mold Shape

**Figure 7:**
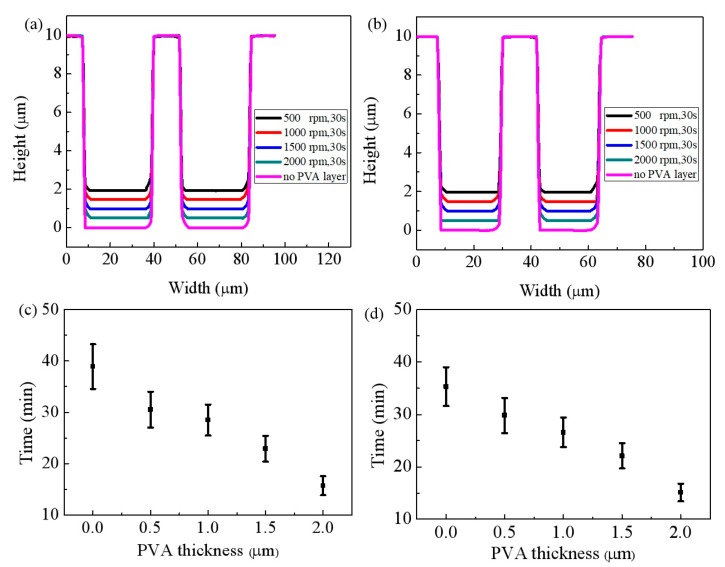
Influence of different mold shapes: (**a**,**b**) Surface profiles of 60 μm × 30 μm size and 60 μm × 20 μm size PDMS molds after spin-coating PVA at different speeds; (**c**,**d**) the release time of PCLC flakes in 60 μm × 30 μm size and 60 μm × 20 μm size PDMS molds with different PVA layer thicknesses.

For different applications, the requirements for the PCLC flake size are also different. For example, the size of PCLC flakes influences the electric driving property of an electricity responsive device [8]. Therefore, we also studied the influence of PDMS mold shape. Three different mold shapes (60 μm × 60 μm, 60 μm × 30 μm and 60 μm × 20 μm) were selected to study the influence of mold shape. The surface profiles of the PDMS molds with different shapes after spin-coating PVA at different speeds are shown in Figure 6a, Figure 7a, and Figure 7b. From the results, we can see that the PVA layer thickness is not be influenced by the mold shape and is only influenced by the spin-coating speed. The release time of the PCLC flakes in the PDMS molds with different shapes are shown in Figure 6b, Figure 7c, and Figure 7d. From the result, we can see that in all kinds of mold, the release time is shorter when the PVA layer is thicker. At the same time, the release time is also shorter when the mold is narrower and smaller. For flakes made using a 60 μm × 20 μm size mold with a 2 μm PVA layer, the release time is less than 15 min. However, the influence of the mold shape is not very significant; the difference between release times with different molds is only 30%, while the difference in release time with different PVA layer thicknesses can reach 67%. 

## 4. Conclusions

Since each PCLC flake can selectively reflect light, it holds promise as an alternative display technology in flexible, reflective displays and other applications such as tunable infrared reflectors. In this paper, we have demonstrated a simple technique for manufacturing shaped PCLC flakes of different sizes using soft lithography. A flexible PDMS mold was made as an inverse replica of silicon wafers patterned with different features. In order to release the PCLC flakes without breaking them and to be able to reuse the PDMS mold, a PVA layer was coated upon the PDMS mold which helped to release the PCLC flakes easier and faster. The alignment and morphology of PCLC flakes made by this method is quite good and the reflectance of the flakes of full size can reach 50%. The thickness of the PVA layer will influence the reflectance and release time of the PCLC flakes. When the PVA layer is thicker, the reflectance of the PCLC flakes is lower and the release time is shorter. The shape of the PDMS mold can also influence the release time of PCLC flakes, but not significantly.

## Supplementary Materials

The following are available online at https://www.mdpi.com/2072-666X/10/7/441/s1, Figure S1: DSC result of the monomer mixture.
