# Supplementary material for: Optimized Soft Lithography Method for Polymer Cholesteric Liquid Crystal Flakes Fabrication
Source: Micromachines (Basel). 2019 Jul 1;10(7):441. doi: 10.3390/mi10070441 (PMC6680817; doi:10.3390/mi10070441)
Supplement: Supplementary file 1 [file micromachines-10-00441-s001.pdf]

micromachines

Supplementary Materials

Optimized Soft Lithography Method for Polymer Cholesteric Liquid Crystal Flakes Fabrication

Guanqing Zhou [1,3,†], Sunqian Liu [1,2,†], Wei Liu [1,2], Dong Yuan [1,2,*] and Guofu Zhou [1,2]

1. Guangdong Provincial Key Laboratory of Optical Information Materials and Technology & Institute of Electronic Paper Displays, South China Academy of Advanced Optoelectronics, South China Normal University, Guangzhou 510006, China
2. SCNU-TUE Joint Lab of Device Integrated Responsive Materials (DIRM), National Center for International Research on Green Optoelectronics, South China Normal University, No 378, West Waihuan Road, Guangzhou Higher Education Mega Center, 510006 Guangzhou, China
3. Department of Physics and Astronomy and Collaborative Innovation Center of IFSA (CICFSA), Shanghai Jiao Tong University, Shanghai 200240, China

† These authors contributed equally to this work and should be considered co-first authors

* Correspondence: yuandong@scnu.edu.cn

Received: 27 May 2019; Accepted: 25 June 2019; Published: 1 July 2019

DSC result of the LC monomer mixture

The phase transition temperatures of the LC monomer mixture were studied by Differential Scanning Calorimeter (DSC, METTLER DSC 1, Mettler Toledo, Inc., Zurich, Switzerland). The DSC result shows that the mixture was in the cholesteric phase in the temperature range between 40 °C and 65 °C, as shown in Figure S1. At higher temperatures it became isotropic.

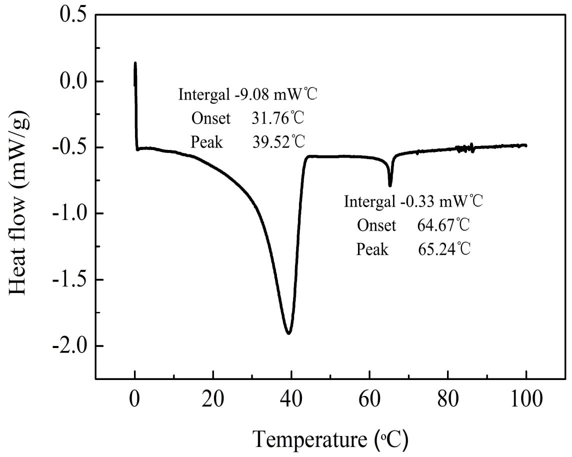

Figure 1. DSC result of the monomer mixture.
